# Flat Feline Faces: Is Brachycephaly Associated with Respiratory Abnormalities in the Domestic Cat (*Felis catus*)?

**DOI:** 10.1371/journal.pone.0161777

**Published:** 2016-08-30

**Authors:** Mark J Farnworth, Ruoning Chen, Rowena M. A. Packer, Sarah M. A. Caney, Danièlle A. Gunn-Moore

**Affiliations:** 1 School of Biological Sciences, Plymouth University, Devon, United Kingdom; 2 The Royal (Dick) School of Veterinary Studies and The Roslin Institute, University of Edinburgh, Edinburgh, United Kingdom; 3 Department of Clinical Science and Services, Royal Veterinary College, Hatfield, Hertfordshire, United Kingdom; 4 Vet Professionals Limited, Midlothian Innovation Centre, Pentlandfield, Roslin, Midlothian, United Kingdom; University of Bari, ITALY

## Abstract

There has been little research into brachycephalism and associated disorders in cats. A questionnaire aimed at cat owners was used to determine the relationship between feline facial conformation and owner-reported cat management requirements and respiratory abnormalities. Owner-submitted photographs of cats were used to develop novel measures of skull conformation. One thousand valid questionnaires were received. Within these there were 373 valid photographs that allowed measurement of muzzle ratio (M%) and 494 that allowed nose position ratio (NP%). The data included 239 cats for which both measurements were available. Owners reported lifestyle factors (e.g. feeding type, grooming routine, activity level), physical characteristics (e.g. hair length) and other health characteristics of their cat (e.g. tear staining, body condition score). A composite respiratory score (RS) was calculated for each cat using their owner’s assessment of respiratory noise whilst their cat was asleep and then breathing difficulty following activity. Multivariate analyses were carried out using linear models to explore the relationship between RS and facial conformation, and lifestyle risk factors. The results showed that reductions in NP% and M% were significantly associated with RS (P < 0.001 and P = 0.026, respectively) and that the relationship was significantly negatively correlated (r = -0.56, P < 0.001 for both). Respiratory score was also significantly associated with increased presence of tear staining (P < 0.001) and a sedentary lifestyle (P = 0.01). This study improves current knowledge concerning cats with breeding-related alterations in skull confirmation and indicates that brachycephalism may have negative respiratory implications for cat health and welfare, as has been previously shown in dogs.

## Introduction

Brachycephalic obstructive airway syndrome (BOAS) is a chronic respiratory condition that arises as a consequence of artificial selection for a changed skull shape primarily characterised by a shortened muzzle [1]. It has been documented as a breed-related disorder in domestic dogs e.g. the pug and bulldog breeds (*Canis familiaris*) [2] and domestic cats e.g. Persian and exotic breeds (*Felis catus*) [3]. In dogs, BOAS has been identified as having the potential to reduce an animal’s quality of life [4] and lifespan [5].

Anatomical abnormalities associated with the brachycephalic conformation were first reported in dogs in 1957 [6] and include stenotic nares, under-sized nasal chambers and an elongated soft palate. These abnormalities restrict airflow and manifest as abnormal and increased respiratory noise (stertor/stridor), dyspnoea, exercise intolerance and, in severe cases, cyanosis and syncope. Since then, research has focused on brachycephalic dogs and has revealed a number of associated respiratory [2, 5] and ocular [7] problems, the risk of which increase with shortening muzzle length, as well as substantial gastro-intestinal and thermoregulatory issues [4]. Other craniofacial health problems associated with brachycephaly in dogs have been less rigorously studied. These include, skin fold dermatitis associated with the pronounced skin folds [8], and dental disease associated with misalignment or overcrowding of the jaw due to changes in its morphology [9]. Such issues may also be prevalent, yet underexplored, in brachycephalic cats. The welfare of brachycephalic dogs has been an area of concern for a wide variety of stakeholders over the last decade, reflected in several high-profile reports including those from the Royal Society for the Prevention of Cruelty to Animals (RSPCA) [10], the Associate Parliamentary Group for Animal Welfare [11], and the Independent Inquiry into Dog Breeding [12]. Given this recent interest, research methods and diagnostic approaches for BOAS in dogs have been improving [2, 7, 13, 14], with computed tomography of the canine skull able to give a better understanding of the anatomical abnormalities present [15]. A more substantial understanding of the primary and secondary anatomical abnormalities that result in BOAS has also, in turn, lead to the development of surgical interventions to alleviate the condition [4].

However, little work has focussed on brachycephalic cats and associated health problems. As a result of this disparity it remains unclear whether brachycephalia manifests and impacts differently in cats as compared to dogs [16]. From the relatively scant literature addressing brachycephalism in cats, it has been demonstrated that selection for shorter skulls with reduced nasal bones is associated with an increased likelihood of the cats suffering from respiratory and other health problems [17, 18]. The severity of feline brachycephalic conformation can vary from mild or moderate, to profound and extreme in accordance with the extent of the skull malformation [18]. Among the brachycephalia-induced health problems affecting cats, BOAS prevalence appears to be relatively underexplored but is anecdotally common. Similar to dogs, anatomical changes in the feline upper respiratory tract include stenotic nares, an elongated soft palate [19] and compressed nasopharyngeal turbinates [20], which increase airway turbulence and resistance, inducing stridulous breathing [21]. In severe cases brachycephalia-related anatomical changes may result in laryngeal trauma, inflammation and associated swelling, laryngeal collapse, nasopharyngeal stenosis, and upper airway swelling; as for dogs surgical procedures may be required to alleviate the problems [19].

Previous reports indicate that over half (58%) of brachycephalic dog owners failed to recognise the breathing abnormalities in their own dog as a health problem [22], which can have a detrimental impact on their dog’s welfare due to under treatment. Due to the growing demand for brachycephalic cats [23], it is necessary to consider if and how cats are affected by extreme skull conformation and whether their owners’ are able to identify any associated negative effects and, where necessary, seek treatment. With this in mind the aims of this research were to:

1. Identify statistical associations between novel measures of feline skull conformation and the occurrence of owner-reported respiratory abnormalities; and
2. Identify relationships between brachycephalism and routine management and care provided by owners.

## Materials and Methods

### Survey design

An online questionnaire for cat owners was constructed (S1 Appendix) with reference to previous research on brachycephalism in dogs [22, 24]. If there was more than one cat within the household a response was only requested for the eldest cat to improve independence of individuals in the statistical analysis. The questionnaire included 30 questions divided into sections which addressed: general owner and cat details; the cat’s health characteristics; and general cat care and owner attitudes (the data on the latter aspect will be considered elsewhere). The assessment of body shape was conducted using the Body Condition System developed by Nestlé PURINA^®^ [25]. Owners were asked to choose between five standard images (from ‘very thin’ to ‘obese’) as to which best matched their cat. Frequency of increased respiratory noise (snorting, snoring or wheezing) in the cat whilst asleep was assessed using a 4-point scale from very quiet (i.e. none) to almost continuous. Experience of respiratory difficulty following activity was explored using a 6-point scale (more than once per day, daily, weekly, monthly, rarely, never). Respondents were also asked to report previous medical diagnoses for their cat. Questionnaires which were not fully completed were excluded from the analyses. Absence of uploaded photographs was not considered to affect completeness of the submitted survey.

Owners were asked to upload recent photographs of their cats, including exact face-on and side-on aspects that included the chin and the ears. Examples of the type of photographs needed were provided as guidance. These photographs provided objective information for measurements of feline skull conformation (Section 2.3). Following visual inspection, photographs were excluded if they did not conform to the required orientation or lacked inclusion of the chin and ears.

### Recruitment of cat owners

Recruitment was achieved entirely via online questionnaire distribution using an English version on the VetProfessional website (www.catprofessional.com) and a Chinese translation of the survey on the Chinese survey website (www.wenjuan.com). In the UK, the English version of the questionnaire was also linked to the websites of International Cat Care (http://icatcare.org/) and Cats Protection (http://www.cats.org.uk/). Responses were anonymous and respondents were informed that completion and submission of the survey were considered as provision of consent for the information to be used for the purposes of research and publication. Respondents could exit the survey at any time without submitting their data.

### Calculation of respiratory scores (RS) and collection of morphometric data

**Respiratory scores (RS)** were created for each cat using the owner’s assessment of respiratory noise whilst the cat was asleep (scale 1 = very quiet; 4 = almost continuous snoring/snorting/wheezing) and the breathing difficulty following activity scale (1 = never; 6 = more than once per day). Owners were asked not to assess their cat’s respiratory noise when it was purring as this showed their cat was not completely asleep and gave erroneous values. The RS was calculated by adding the two scores, with the total on a scale from two to 10; an increased score represented more substantial owner-reported respiratory noise and difficulty.

**Fig 1:**
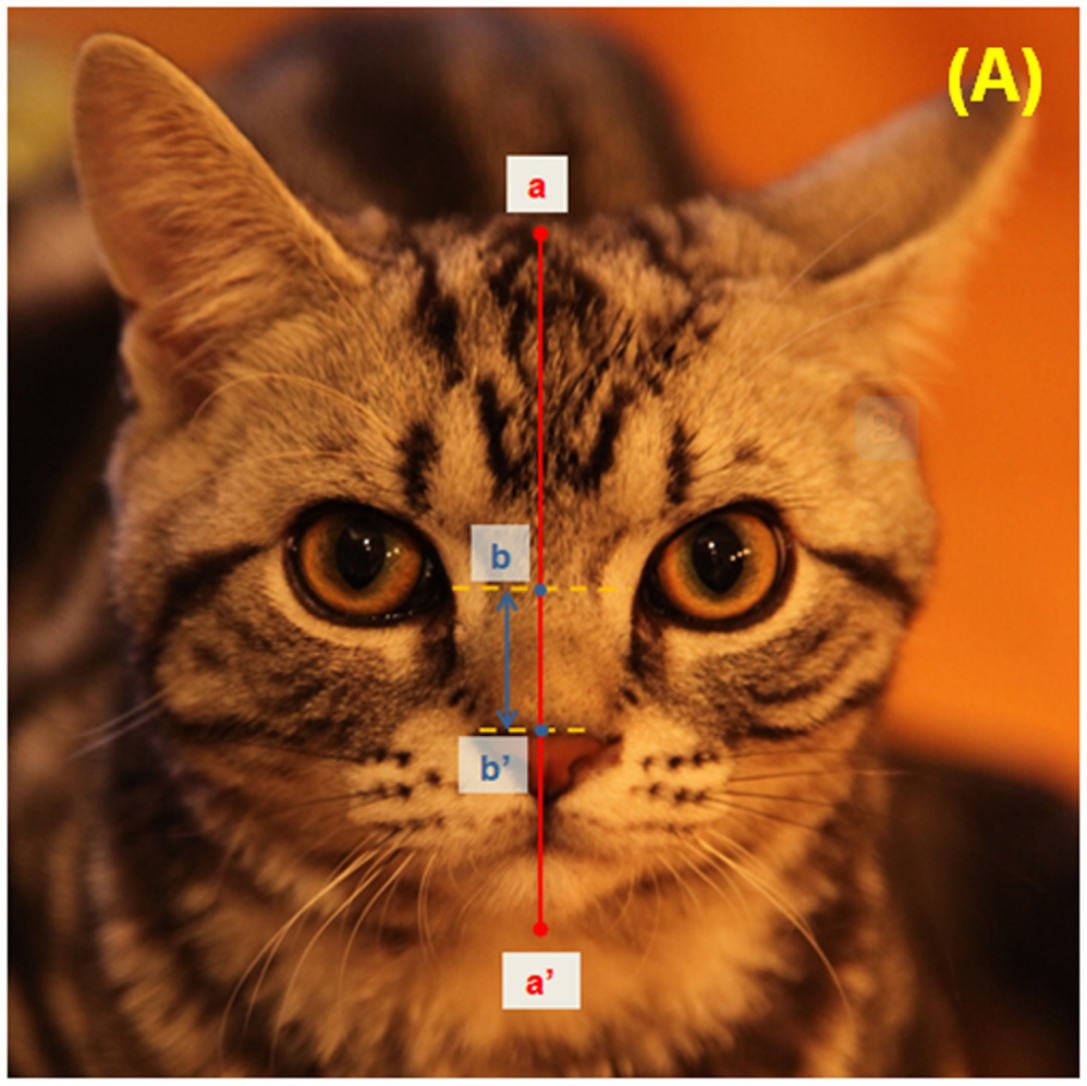
Calculation of proportional nose position ratio (NP%). The length of foreface is defined as the maximum distance (mm) of facial length (a-a’ shown as a red line). The eye-nose distance is defined as the straight-line distance (mm) between the midpoint of the medial canthus (equivalent to the nasal stop on the lateral view) and dorsal tip of nose (b-b’, shown in blue line). If the dorsal tip of the nose is in line with the midpoint of the medial canthus the distance is given a zero value, deviation to above this point is provided a negative value. For this picture NP% = 17.6% ([eye-nose distance 12mm / length 68mm] *100). Note for readers: measurements will differ based on the size of viewed image.

**Fig 2:**
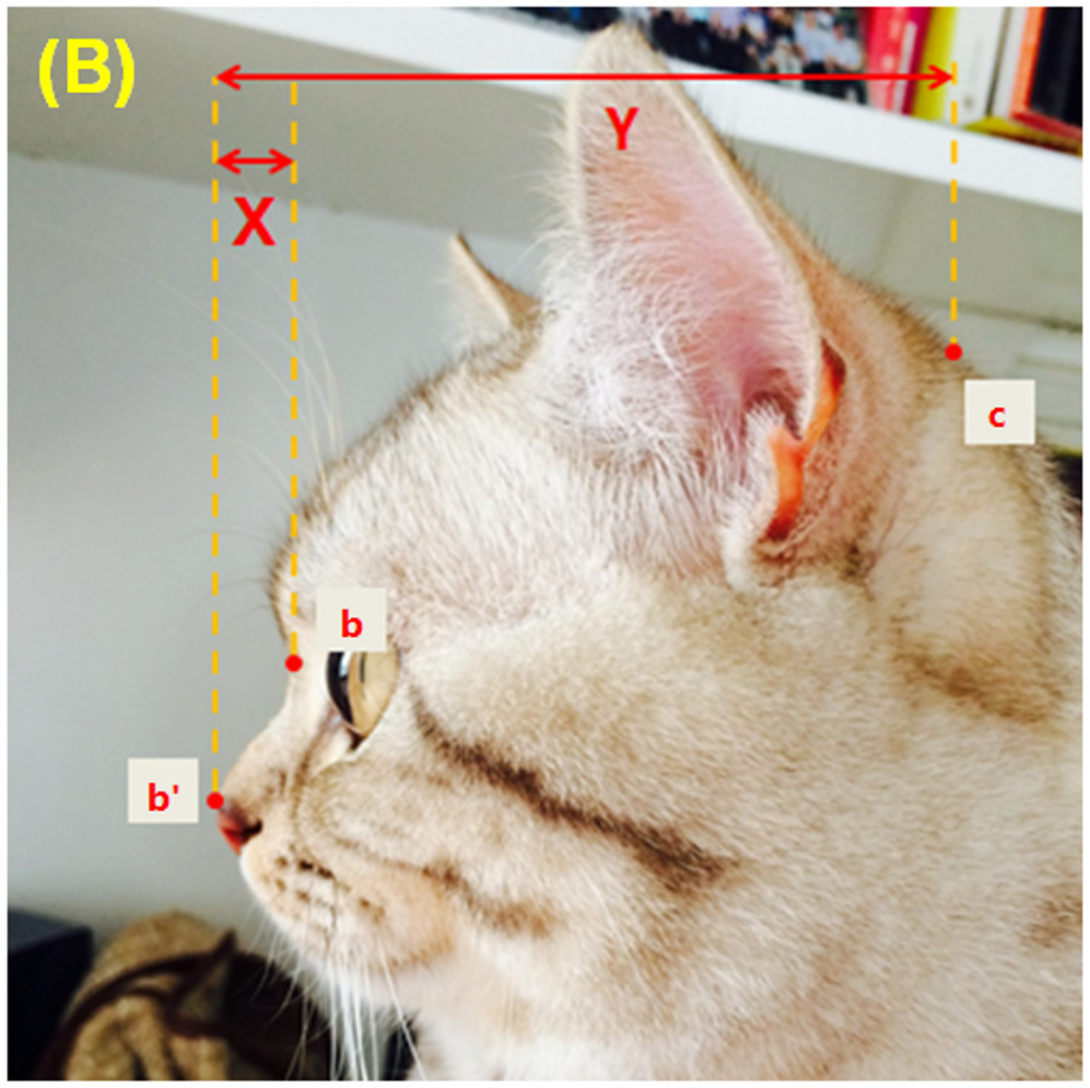
Calculation of Proportional muzzle length ratio (M%). Cranial length is defined as the horizon-line distance (mm) from the dorsal tip of the nose to the occipital protuberance (b’-c). Muzzle length is the horizon-line distance (mm) from the dorsal tip of nose to the nasal stop (b’-b). In the event that the dorsal tip of the nose is in line with the nasal stop a zero value is assigned. For this picture M% = 7.89% ([muzzle length 6mm / cranial length 76mm] *100). Note for readers: measurements will differ based on the size of viewed image.

Facial photographs were used to quantify skull conformation. The measurement protocol quantified the following conformational features: (i) foreface length and (ii) eye-nose distance from frontal photographs (Fig 1); (iii) muzzle length and (iv) cranial length from profile photographs (Fig 2). All measurements were taken to the nearest millimetre, using bony landmarks as set points on the skull to ensure the consistent application of measures across divergent skull morphologies.

#### Calculation of the nose position ratio (NP%)

This is calculated by dividing the eye-nose distance by the foreface length and multiplying by 100 (see Fig 1): If the dorsal tip of the nose was in line with the midpoint of the medial canthus the distance was given a zero value, deviation to above this point was provided a negative value.

#### Calculation of muzzle ratio (M%)

This is calculated by dividing muzzle length by cranial length and multiplying by 100 (see Fig 2): Cranial length was assessed using the horizon-line distance (mm) from the dorsal tip of the nose to the occipital protuberance. Muzzle length was the horizon-line distance (mm) from the dorsal tip of nose to the nasal stop. In the event that the dorsal tip of the nose is in line with the nasal stop a zero value is assigned.

Two independent investigators were recruited to assist author RC in assessing all the cat photographs. Three independently measured datasets were created and the mean value for each measurement was then used as the final morphometric datum point.

### Ethics statement

This study was approved by The Royal (Dick) School of Veterinary Studies Ethics in Research Committee (Human).

### Data analyses

For the 1000 completed responses, cats were allocated as either likely to be brachycephalic (BC) or non-brachycephalic (NBC) based on owner report of the breed and the breed standard. This was only used for analyses that pertained to overall consideration of tear staining and cat activity. To avoid bias (based on incorrect owner report) all other analyses used objective measurements of the degree of brachycephalism from owner supplied pictures; these were assessed independently of allocation to the NBC or BC category. Data (S1 Dataset) were analysed in IBM SPSS Statistics v21. All data were inspected visually using histograms to determine the distribution of the data, and normally and non-normally distributed variables were analysed appropriately. Univariate analyses were used to identify associations to take forward into a multivariate analysis (if p<0.02). Chi-squared tests were used for categorical responses and t-tests and Spearman’s Rank Correlation tests for continuous responses. Multivariate analyses were carried out using linear models. Respiratory score was the continuous response variable in all models. Relevant morphometric predictors significant at the univariate level (M% and NP%) were modelled as continuous fixed effects, and physical predictors (e.g. hair length and body condition score), health predictors (e.g. tear staining) and lifestyle predictors (e.g. food type, activity level) as categorical fixed factors. Multi-collinearity was checked for in all models, identified from inflated standard errors in the models, and thus avoided. Model fit was assessed using the deviance and Akaike's information criterion. Due to collinearity between the breed-type category of brachycephalic [i.e. Persian and exotic breeds] vs. non-brachycephalic cats and M% and NP% variables, both variables could not be used in the same model. As M% and NP% were objective measures of conformation in comparison to the relatively subjective breed-type categorisation (based on breed as reported by the owner) they were preferentially kept in the models. This also prevented unintentional exclusion of non-purebred cats with moderate brachycephalia (e.g. first generation crosses) from comparisons. Two way mixed intra-class correlation (ICC) absolute agreement analyses were performed to establish inter-rater reliability for the morphometric data using the NP% measurements. P<0.05 was considered significant.

## Results

### Response information

Within a two-month period from April to June 2015, 1,511 cat owners replied to an online survey, with the 1,000 (66.2%) completed responses retained for further analyses. Of these, 40.5% (405/1000) were from China, 44.0% (440/1000) United Kingdom and 15.5% (155/1000) from other countries, including the United States of America, Australia, New Zealand and other European countries. Of the responses, 53% were for male cats (531/1000). Cats reported as purebred accounted for 30% (122/405) of responses from China and 37.6% (165/440) of responses from the UK (Table 1). From the 1000 responses, 494 valid photographs were provided that allowed for the NP% calculation (Fig 1) and 373 valid photographs allowed for the M% calculation (Fig 2). Of these 239 cats had both aspects supplied.

**Table 1:** Breed information for purebred cats from the United Kingdom (n = 165) and China (n = 122), as reported by respondents to an on-line survey.

Reported Breed	China	United Kingdom (UK)
**British Shorthair**	34	25
**Exotic**	30*	-
**Persian**	19**	14***
**Scottish Fold**	11	-
**Siamese**	9	13
**Ragdoll**	5	20
**British Blue**	4	1
**Shandong Lion Cat**	3	-
**Norwegian Forest Cat**	2	4
**Oriental Shorthair**	2	6
**Maine Coon**	1	12
**Birman**	1	10
**Abyssinian**	-	4
**American Shorthair**	-	4
**Australian Mist**	-	3
**Bengal**	-	17
**Burmese**	-	17
**Devon/Selkirk/Cornish Rex**	-	5
**Tonkinese**	-	4
**Other**	1	4

‘Other’ category includes all reports for a single purebred across all responses. Breeds included are (China) angora; (UK) napoleon, ocicat, sphynx and Turkish Van.

* Exotic includes cats reported as ‘Garfield’ (n = 19) or ‘Iso Short’ (n = 3).

** Persian includes cats reported as ‘Himalayan’ (n = 2) or ‘Chinchilla’ (n = 5).

*** Persian includes cats reported as ‘Himalayan’ (n = 1) or ‘Chinchilla’ (n = 3)

### Factors associated with Respiratory Scores

**Fig 3:**
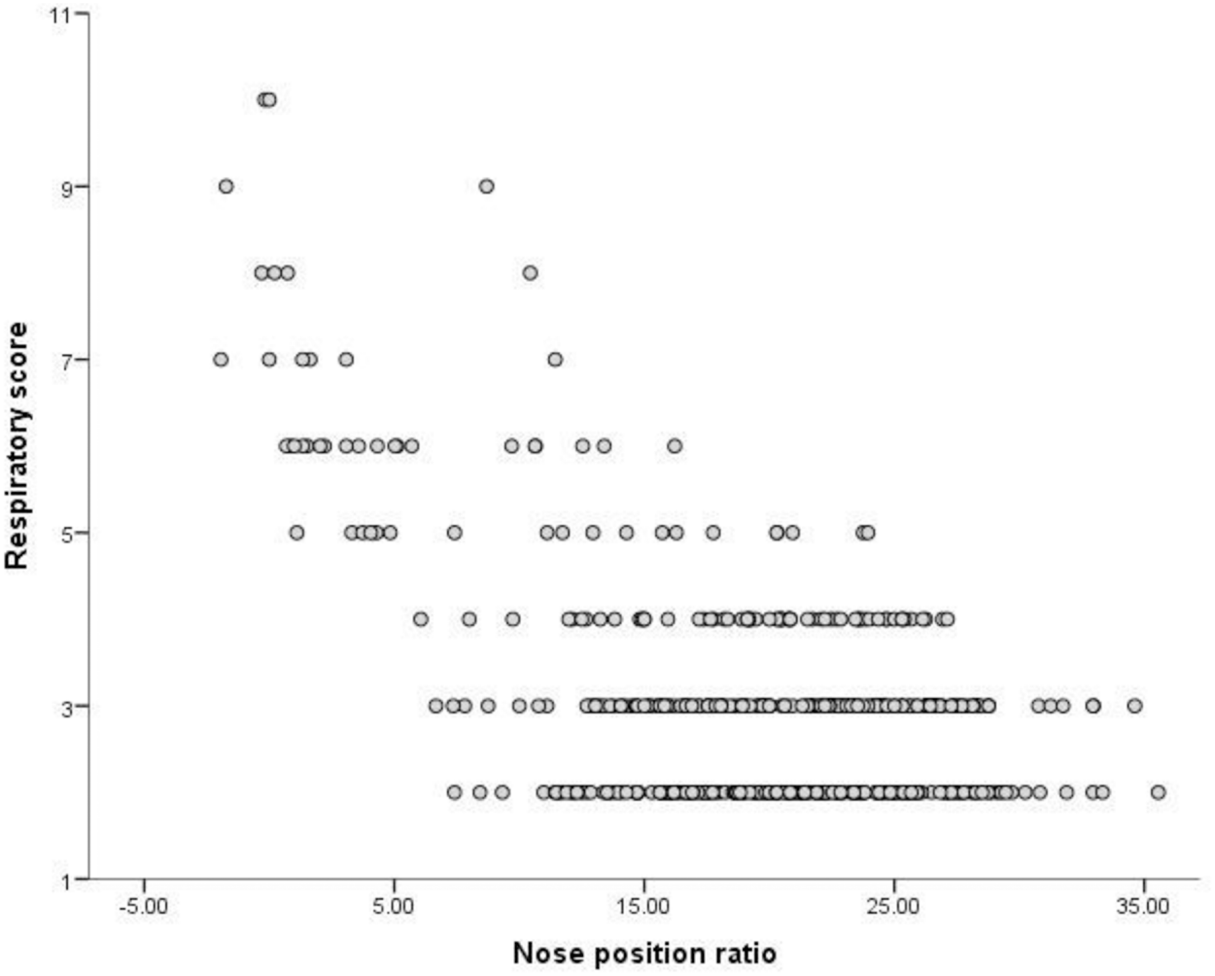
Correlation between proportional nose position (NP%) and respiratory score (RS) calculated from owner-reported respiratory noise for companion cats (r = -0.56, p<0.001, n = 494).

**Fig 4:**
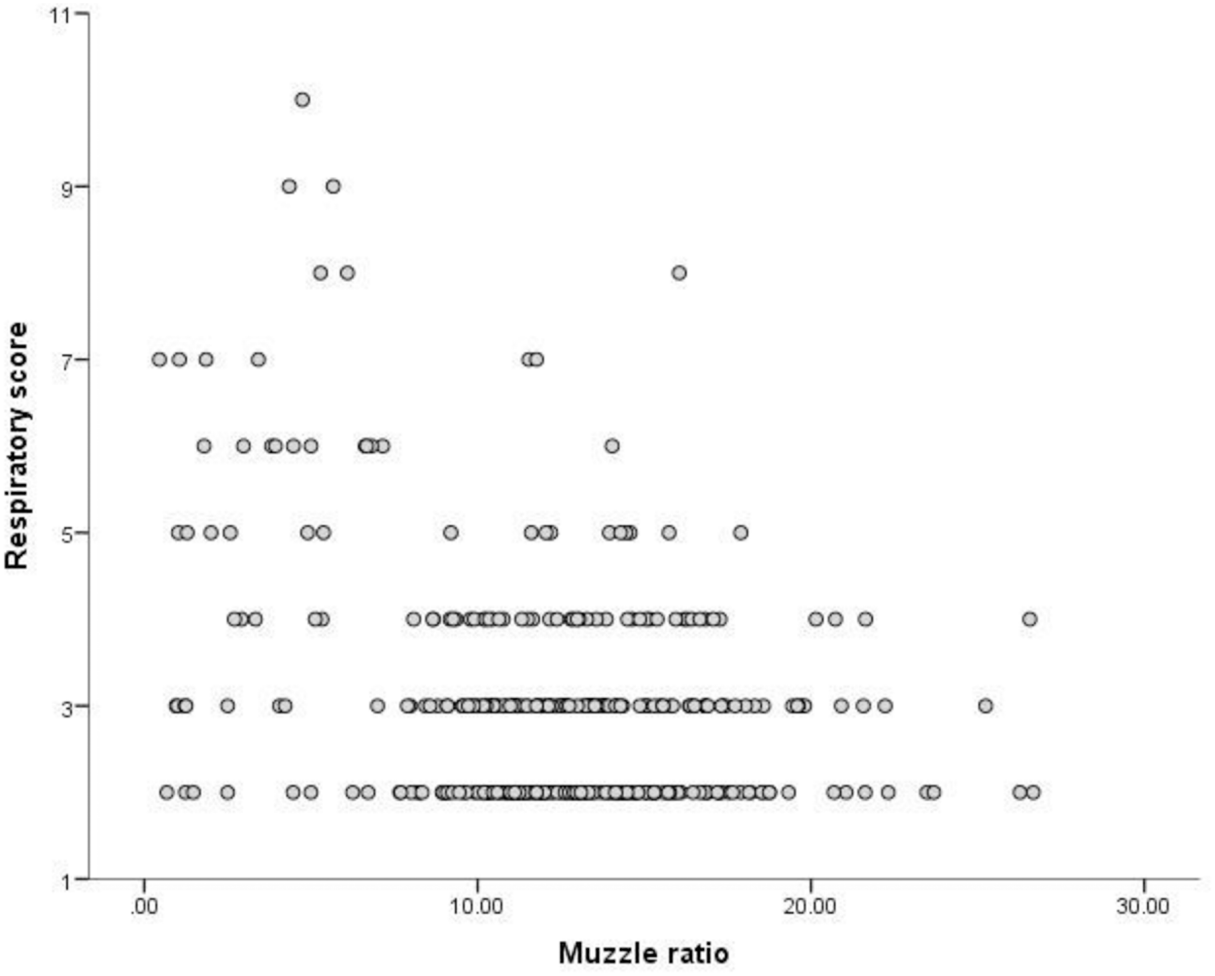
Correlation between proportional muzzle ratio (PML) and respiratory score (RS) calculated from owner-reported respiratory noise for companion cats (r = -0.56, p<0.001, n = 373).

Univariate exploratory analyses identified significant associations between RS and NP%, M%, level of activity and tear staining (all *P*<0.0001), BCS (*P* = 0.024), hair length (*P* = 0.003) and feeding wet food (e.g. pouches or tins) (*P* = 0.046). The presence of tear staining and decreasing levels of activity were both associated with an increased RS (Table 2). Significant negative associations were found between NP% and M% (NP%: r = -0.56, p<0.001, n = 494;M%: r = -0.56, p<0.001, n = 373), with lower M% and NP% ratios being associated with higher RS (Figs 3 and 4).

**Table 2:** Descriptive statistics of categorical variables significantly associated with respiratory score as calculated for 1000 cats from owner-reported responses concerning breathing noise whilst asleep and breathing difficulty following activity.

Categorical Variable	Sub-category	Mean Respiratory Score (95% Confidence interval)	SD	N
**Tear staining**	No	2.77 (2.70–2.84)	1.00	759
	Yes	3.54 (3.33–3.74)	1.63	241
**Activity level**	Sedentary	3.25 (3.07–3.43)	0.09	264
	Adequate	2.94 (2.83–3.04)	1.14	452
	Active	2.78 (2.64–2.92)	1.03	214
	Very active	2.50 (2.23–2.77)	1.15	70

These factors were therefore included in the multivariate analysis. Significant associations (see Table 3) were retained between RS and M% (P = 0.026); NP% (P < 0.001); tear staining (P < 0.001) and activity level (P = 0.01).

**Table 3:** Multivariate model of the relationship between respiratory score (RS) and skull conformation, health and lifestyle variables.

Variable	Sub-category	Coefficient	SE	t	P
**Muzzle ratio (M%)**	*Continuous*	-0.04	0.017	-2.25	0.026
**Nose position ratio (NP%)**	*Continuous*	-0.07	0.012	-6.21	<0.001
**Tear staining**	No	-0.91	0.191	-4.77	<0.001
	Yes	*Reference*			
**Activity level**	Sedentary	0.75	0.28	2.61	0.010
	Adequate	0.26	0.26	0.97	0.332
	Active	0.17	0.28	0.60	0.547
	Very active	*Reference*			

Due to the association between tear staining and RS, the relationships between tear staining and conformation measures (i.e. M% and NP%) were also analysed to examine whether this was a confounding factor that may explain this relationship. There was a significant difference in both M% and NP% between cats with or without tear staining (Muzzle length: Staining = 10.18 (±4.91); No staining = 12.98 (±4.33); Mann-Whitney = 12934, p<0.001); Nose position ratio: Staining = 16.43 (±8.81); No staining = 20.40 (±5.85); Mann-Whitney = 16735, p<0.001) with cats with tear staining having lower M% and NP% ratios.

### Inter-rater reliability

All three independent raters measured 100% of the photographs associated with NP% (n = 493). The ICC for calculation of NP% indicated high inter-rater reliability (0.793 [95% CI: 0.757–0.824]).

## Discussion

Although overall RSs for cats in this study were low, indicating that the majority of owners reported few breathing abnormalities in their cats, this study provides evidence that skull confirmation does have a significant impact upon owner reports of increased respiratory difficulty and/or noise. Prior to this study there was little literature focusing on the definition of the feline brachycephalic head conformation, or the potential health risks that may be associated with it. What had been published had often used *post-mortem* techniques (anatomical dissection and corrosion casting) [26], radiography [27], or advanced imaging (helical computed tomography [CT] with three-dimensional [3D] constructs) [18]. Although using these techniques enables precise measurements of the animals’ skulls, they are costly, require sedation/anaesthesia and may be inconvenient, and thus are not feasible for large-scale data collection. The non-invasive and simply applied measurement protocols proposed in this study could be useful when gathering information for ongoing research concerning brachycephalism in cats. They may also prove useful to veterinarians or owners for diagnostic purposes or during health checks, and breeders may wish to consider them when making breeding decisions or interpreting breed standards.

The two conformational measurements (NP% and M%) were both significantly associated with RS; however, it was evident that the most significant effect was the cat’s nose position, as quantified by NP%. A smaller NP% predicted a higher risk of breathing difficulties; this relationship can be explained by abnormalities of the upper respiratory system such as increased soft tissue within the nose, laryngeal pathology and/or soft palate dysgenesis, all of which can be associated with brachycephalic head conformation [19]. More caudally positioned noses, that are closer to the eyes, may be associated with the nasal turbinate bones being displaced into the nasopharynx (nasopharyngeal turbinates) [20] increasing airway resistance and thus increasing the respiratory score. In brachycephalic dogs, respiratory compromise is often associated with elongation of the soft palate, which is seen in ≥85% of BOAS cases [4]. An informal search of the literature identifies that soft palate elongation is rarely documented in brachycephalic cats. It is unclear whether this is because it may contribute to a lesser extent to BOAS in cats compared with dogs or whether it is simply underexplored or under-recognised. Further study of brachycephalism-induced conformational changes in cats is required, with the external measurements explored here investigated for their association with internal airway abnormalities.

From these data it follows that brachycephalic breed-types such as Persians or Exotics are more likely to have respiratory difficulties and/or substantial breathing noise, as compared to other breed-types. This agrees with the findings of similar research on brachycephalic dogs [2, 28].

According to Malik *et al*. [21] and Schlueter *et al* [18], the presence of tear staining is induced by the obstruction of nasolacrimal system, blocking draining and causing tears to flow down the face. Thus, with increasing brachycephaly associated with obstruction to both the airways and nasolacrimal system, it is unsurprising that an increased RS is concomitant with an increased likelihood of the presence of tear-staining (Table 2).

Degree of activity was negatively associated with an increased RS, with sedentary cats receiving the highest scores. As a high RS is likely indicative of increased respiratory obstruction, it is possible that dyspnoea associated with increased activity in these cats’ results in more sedentary behaviour as they are incapable of high activity levels. However, it is reasonable to assume that some sedentary cats may not have received an entirely indicative RS because a lack of activity reduces the likelihood of activity-induced changes in respiration, and thus their RS score may be underestimated. Given the relationships between these variables and the RS, it follows that cat-based and owner-based variables can be regarded as exacerbating or indicative factors of brachycephalism-induced problems.

The measurement methods used in this study have some limitations, as they have a degree of subjective visual assessment (e.g. the influence of long fur obscuring the exact top of the head [Fig 1] and/or the occipital protuberance [Fig 2]) which may impact upon the reliability of data measurement. Although the data sets of skull conformation came from the mean of three investigators, measurement error was still evident as shown by the ICC for NP%. However, given the novelty of the process and the general congruity of the results, it is felt that this method is worthy of greater investigation. It remains important to ensure that measurements are made effectively by providing instruction in taking measurements and making assessments between raters.

The RS in this study was based on the information cat owners reported. The method could benefit from clinic-based verification to establish if veterinarian-assessed severity of breathing-related issues in brachycephalic cats concurs with owners’ subjective recognition and assessments. That said, clinic-based verification may not completely agree with the RS generated here as the first part of the RS was generated by observing the cat’s breathing when it was asleep. The RS used here is a simplified version of the owner reported breathing score (for dogs) used elsewhere [22]. It could therefore benefit from more nuanced questions concerning respiratory noise and breathing difficulty in more varied scenarios; however, accurate appreciation of additional active scenarios may be challenging for owners with primarily outdoor or excessively sedentary cats.

## Conclusion

This study demonstrated that owner-reported respiratory difficulties and abnormal breathing sounds are significantly affected by the facial conformation of cats, such that shorter muzzles were associated with more respiratory compromise. Being able to make non-invasive measurements of brachycephalism may provide practitioners and researchers with a valuable tool to further investigate this phenomenon. Attention should be paid to breed standards which promote increased brachycephalia in cats which has the potential to negatively impact upon their welfare, and potential buyers of brachycephalic cat breeds should be made aware of the risks of their conformation.

## Supporting Information

S1 Appendix Complete survey to explore cat-owner attitudes and experiences. (DOCX)

S1 Dataset Respiratory scores (RS) were created for each cat using the owner’s assessment of respiratory noise whilst the cat was asleep (scale 1 = very quiet; 4 = almost continuous snoring/snorting/wheezing) and the breathing difficulty following activity scale (1 = never; 6 = more than once per day). Food is whether or not cats were fed a wet food diet. Breed was attributed as brachycephalic or non-brachycephalic based on owner-reported breed. Body Condition Score (BCS) was based on Body Condition System developed by Nestlé PURINA^®^.
(XLSX)
