# Supplementary material for: Flat Feline Faces: Is Brachycephaly Associated with Respiratory Abnormalities in the Domestic Cat (Felis catus)?
Source: PLoS One. 2016 Aug 30;11(8):e0161777. doi: 10.1371/journal.pone.0161777 (PMC5004878; doi:10.1371/journal.pone.0161777)
Supplement: S1 Appendix — (DOCX) [file pone.0161777.s001.docx]

**Appendices**

**Appendix 1**

**Cat Life Style & Face Shape Survey**

This survey is concerned with the general health and lifestyle of cats. It also includes questions on your thoughts about different breeds of cats and their risk of developing illness. To help us gain more detailed results we ask you to upload photographs of your cat; this is optional and none of the pictures will be disseminated publically without permission.

The photos we are looking for are:

- exact face on *
- side of face view including the chin and the ears *
- if possible, a video clip of your cat breathing (10-15 seconds long), but please do not worry if you don't have one

* Please see example pictures below.  You will be prompted to upload the images at Question 30. Remember submitting photos and/or video is optional.

Please complete only ONE questionnaire per household and for the OLDEST cat. We are especially interested in hearing about pedigree cats so if you own a pedigree cat, please complete the survey for this cat. However, if you only own non-pedigree cats, we are still very interested in hearing from you. The survey takes about 20 minutes to complete.

**Thank you very much for your kind co-operation!**


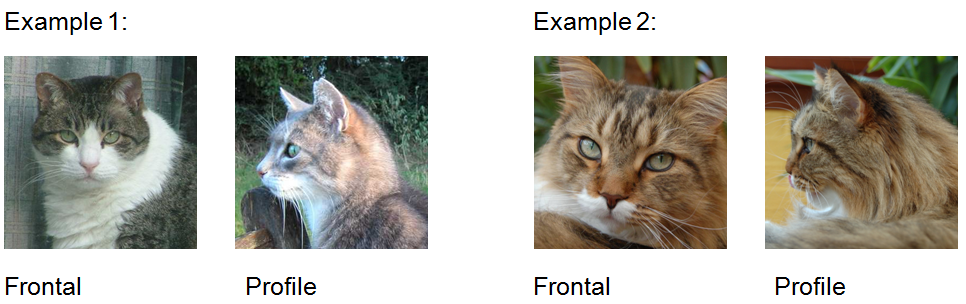


After this survey has finished and the data been analysed, we are happy to provide you with summary feedback. If you wish to receive a summary of our findings please leave your email address (it will be held separately from your responses to the rest of the survey to maintain your anonymity).

|  |
| --- |

Completion and submission of the survey is considered as your consent for us to use the information for the purposes of research and possible publication. You may exit the survey at any point without submission. Your data will be anonymous and you cannot be identified. All data are exclusively for the use of the research group and will not be given to third parties.

**I        General information**

Your Gender *

|  |
| --- |

What country do you live in?

Do you or have you worked in veterinary or allied animal health professions? *

|  |
| --- |

**II       Basic Information about your CAT**

Please complete only ONE questionnaire per household and for the OLDEST cat. We are especially interested in hearing about pedigree cats so if you own a pedigree cat, please complete the survey for this cat. However, if you only own non-pedigree cats, we are still very interested in hearing from you. Thank you!

What is your cat’s name?

|  |
| --- |

1. Breed?

|  |
| --- |

2. Is your cat pedigree registered? *

|  |
| --- |

3. Age (years / months)? *

|  |
| --- |

4. If you know your cat’s weight, please enter it below in kg

|  |
| --- |

5. Gender? *

|  |
| --- |

6. Have you ever bred from your cat? : *

|  |
| --- |

(i) If yes, how many litters have they produced? *

|  |
| --- |

(ii) Would you describe yourself as a cat breeder? *

|  |
| --- |

7.  Have you ever shown your cat competitively? *

|  |
| --- |

(i) If yes, did they gain awards? *

|  |
| --- |

8. Where does your cat usually live? *

|  | 100% Indoors |
| --- | --- |
|  | 100% Free-roaming outdoors |
|  | Mostly (≥75%) Indoors |
|  | Mostly (≥75%) Free-roaming outdoors |
|  | Approximately equal time living indoors and outdoors |
|  | Indoors or in an enclosed outdoor pen or garden |
|  | Other |

9. Do you have other pets at home? *

|  |
| --- |

Number of cats? *

|  |
| --- |

Number of dogs?

|  |
| --- |

What breed(s) of dog do you own? Please list breed(s):

|  |
| --- |

Number of other animals?

|  |
| --- |

**III     Your CAT’s diet**

10. What food type(s) do you usually provide for your cat? (Tick all that apply): *

|  | Dry cat food (complete diet) |
| --- | --- |
|  | Wet cat food (pouches or tins) |
|  | Cooked human food (e.g. fresh chicken, ham, cheese, etc.) |
|  | Raw food (e.g. uncooked fresh chicken, etc.) |
|  | Special home-made food |
|  | Specially formulated cat food (e.g. breed specific, or special veterinary diets for health problems, etc.) * |
|  | Other types of food fed |

If yes, what specially formulated cat food do you feed? *

|  |
| --- |

11. Method of Feeding:

Frequency of Feeding: How many meals/day?

|  |
| --- |

Ad lib (i.e. food is there all the time) *

|  | Yes |
| --- | --- |
|  | No |

Is this cat fed separately from other pets *

|  | Yes |
| --- | --- |
|  | No |

12. Your evaluation of your cat’s appetite:   *

|  | Don’t know |
| --- | --- |
|  | Less than other cats |
|  | About the same |
|  | More than other cats |

13. Do you give any food Supplements: (e.g. Vitamins)  *

|  | Yes |
| --- | --- |
|  | No |

(i) If yes, what kind of supplement do you provide? *

|  |
| --- |

**IV     Life style & Activities**

14. How long is your cat’s coat? *

|  | Long |
| --- | --- |
|  | Medium |
|  | Short |
|  | Sphinx cat |

15. How many times does your cat need to be groomed by you/your groomer?

Often (enter the typical number of occasions per week)

|  |
| --- |

Sometimes (enter the typical number of occasions per year)

|  |
| --- |

16. Do you ever shave your cat or have them shaved? *

|  | No |
| --- | --- |
|  | Only to remove occasional tangles |
|  | Yes, occasionally |
|  | Yes, they have a regular ‘Lion Cut’ or another form of shave |
|  | Yes, for aesthetic reasons |

17. Does your cat regularly have tear stains on his/her face? *

|  | Yes |
| --- | --- |
|  | No |

18. How many times does your cat need to have his/her face wiped?

Very often (enter the typical number of occasions per day)

|  |
| --- |

Often (enter the typical number of occasions per week)

|  |
| --- |

Sometimes (enter the typical number of occasions per year)

|  |
| --- |

Never (please enter '0')

|  |
| --- |

19. How often does your cat need to be bathed?

Often (enter the typical number of occasions per week)

|  |
| --- |

Sometimes (enter the typical number of occasions per year)

|  |
| --- |

Never (please enter '0')

|  |
| --- |

20. Examples of cat’s body shape:


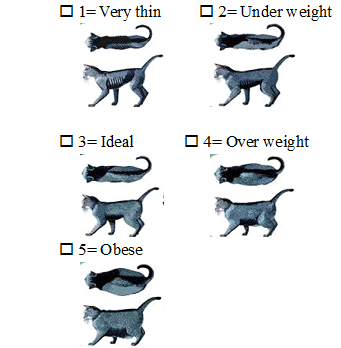


*Pictures courtesy of Nestlé Purina PetCare Company*

20. From the examples above, how do you describe your cat’s body shape?  *

|  | 1 Very Thin |
| --- | --- |
|  | 2 Under Weight |
|  | 3 Ideal |
|  | 4 Over Weight |
|  | 5 Obese |

21. How active is your cat? *

|  | Sedentary (he/she sleeps most of the time) |
| --- | --- |
|  | Adequate (about the same as most other cats) |
|  | Active |
|  | Very active |

22. Do you encourage your cat to exercise? (e.g. play with your cat, buy toys for your cat that they play with, etc.)     *

|  | Yes |
| --- | --- |
|  | No |

23. How long do you spend with your cat every day apart from sleeping time?    *

|  | >16h/day |
| --- | --- |
|  | 12-16h/day |
|  | 7-11h/day |
|  | 3-6h/day |
|  | 0-2h/day |

24. Do you allow the cat to sleep on your bed?   *

|  | Yes |
| --- | --- |
|  | No |
|  | Sometimes |

25. What does your cats purr sound like? *

|  | They don’t purr |
| --- | --- |
|  | Quiet |
|  | Sometimes quiet but other times loud |
|  | Loud |

(i) Does your cat dribble when purring? *

|  | Yes |
| --- | --- |
|  | No |

26. What does your cat’s breathing sound like while asleep? *

|  | Very quiet (other than when purring) |
| --- | --- |
|  | Slight snoring/snorting/wheezing |
|  | Frequent snoring/snorting/wheezing |
|  | Almost continuous snoring/snorting/wheezing |

27. Does your cat sometimes stop breathing while sleeping? *

|  | Yes |
| --- | --- |
|  | No |

**V       Your CAT’s health conditions**

28. How often does your cat show difficulty breathing when engaged in physical activity? (Difficulty breathing could include your cat appearing very short of breath or appearing easily fatigued). *

|  | Never |
| --- | --- |
|  | Rarely |
|  | Monthly |
|  | Weekly |
|  | Daily |
|  | More than once per day |

29. Does your cat currently have breathing problems, or has he/she previously had them? *

|  | Yes |
| --- | --- |
|  | No |

If yes, please answer the following questions:

(i) If known, what was your cat diagnosed with? *

|  |
| --- |

(ii) What treatment (if any) has your cat received for this condition?

|  | Surgery |
| --- | --- |
|  | Medication |
|  | Environmental modification (e.g. changing to dust-free cat litter, not smoking in the house, etc.) |
|  | None |
|  | Other - please fill in any additional information: |

If your cat has previously been treated, how long ago was that treatment? *

|  |
| --- |

(iii) What age was your cat when you first noticed this condition?  *

|  |
| --- |

(iv) What first made you notice this condition? (e.g. change in behaviour, increased breath sounds, collapse, etc.) *

|  |
| --- |

(v) How quickly did these signs appear? *

|  | Suddenly over a few hours |
| --- | --- |
|  | Over a few days |
|  | Gradually over a few weeks |
|  | Gradually over several months |
|  | Other – please state |

(vi) Do you believe this condition is: *

|  | Resolved |
| --- | --- |
|  | Getting better |
|  | Getting worse |
|  | Staying the same |
|  | Comes and goes (but is always there) |
|  | Episodic, they are sometimes free of problem |
|  | Other - please state: |

(i) If episodic, how many episodes have occurred over the recent six months? *

|  |
| --- |

30. We would be really grateful if you would provide ‘ID photos’ of your cat. We need exact face-on and side-of-face views that include the chin and the ears.

We have provided examples of ideal photos as a guide


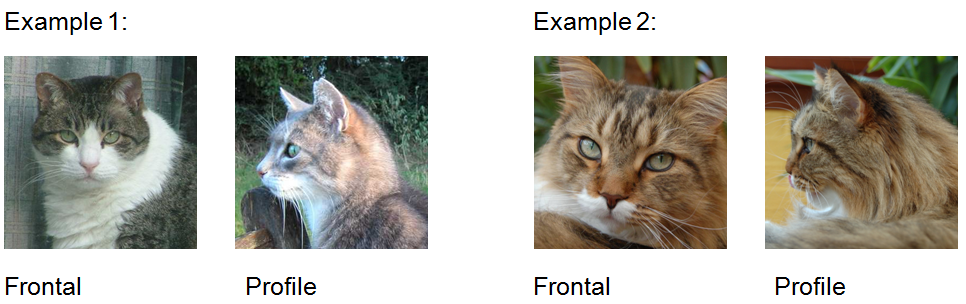


*Pictures courtesy of Danielle Gunn-Moore*

**Image 1**

|  |
| --- |

**Image 2**

|  |
| --- |

32. If you have a video clip or audio recording of your cat breathing, could you please attach it too?

**Please add file/s here:**

|  |
| --- |

**VI Your Perceptions**

33. (i) To what extent do you agree with the following statements?: *

|  | Strongly disagree | Disagree | Neither agree nor disagree | Agree | Strongly agree |
| --- | --- | --- | --- | --- | --- |
| I like short-nosed / flat-faced cats e.g. Persian and Exotic Shorthaired cats, more than any other breeds of cat. |  |  |  |  |  |
| This kind of face is my preferred type as I think they look beautiful |  |  |  |  |  |
| These cats generally have a good temperament. |  |  |  |  |  |
| The short-noses of these breeds may predispose them to some health problems. |  |  |  |  |  |

If there is another reason, please state:

|  |
| --- |

33. (ii)  *

|  | Strongly disagree | Disagree | Neither agree nor disagree | Agree | Strongly agree |
| --- | --- | --- | --- | --- | --- |
| These flat-faced breeds of cats are more vulnerable to breathing distress. |  |  |  |  |  |

33. (iii)  *

|  | Strongly disagree | Disagree | Neither agree nor disagree | Agree | Strongly agree |
| --- | --- | --- | --- | --- | --- |
| These flat-faced breeds of cats have higher probability to suffer from eye or oral disorders.  It is necessary to promote selective breeding of pedigree cat. |  |  |  |  |  |

If you do not agree, please state why:

|  |
| --- |

If you do agree, please state why:

|  |
| --- |

33. (iv)  *

|  | Strongly disagree | Disagree | Neither agree nor disagree | Agree | Strongly agree |
| --- | --- | --- | --- | --- | --- |
| There are health problems related to the face shape of some pedigree cat breeds. |  |  |  |  |  |

33. (v)  *

|  | Strongly disagree | Disagree | Neither agree nor disagree | Agree | Strongly agree |
| --- | --- | --- | --- | --- | --- |
| There are health problems associated with the degree of inbreeding in some pedigree cat breeds. |  |  |  |  |  |

33. (vi) Please feel free to make any additional comments and thoughts about extremely long-faced or short-faced cats:

|  |
| --- |

34. Which face shape do you prefer?  Please mark your preference on the following cats, e.g.   from 0 = don’t like at all; through 5 = neutral; to 10 = this is my favourite type of cat:


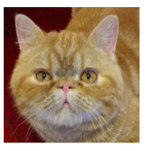

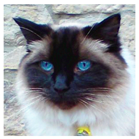

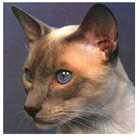


( ) ( ) ( )


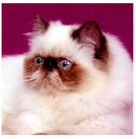

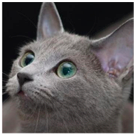

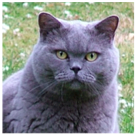


( ) ( ) ( )


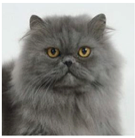

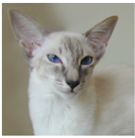

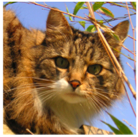


( ) ( ) ( )


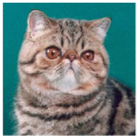


( )

** All photographs were authorized by the owners.*

Thank you for taking the time to complete this questionnaire.
